# Machine learning in onco-pharmacogenomics: a path to precision medicine with many challenges

**DOI:** 10.3389/fphar.2023.1260276

**Published:** 2024-01-09

**Authors:** Alessia Mondello, Michele Dal Bo, Giuseppe Toffoli, Maurizio Polano

**Affiliations:** Experimental and Clinical Pharmacology Unit, Centro di Riferimento Oncologico di Aviano (CRO), Istituto di Ricovero e Cura a Carattere Scientifico (IRCCS), Aviano, Italy

**Keywords:** pharmacogenomics, machine learning, omics, targeted therapy, drug toxicity, drug efficacy, drug repurposing

## Abstract

Over the past two decades, Next-Generation Sequencing (NGS) has revolutionized the approach to cancer research. Applications of NGS include the identification of tumor specific alterations that can influence tumor pathobiology and also impact diagnosis, prognosis and therapeutic options. Pharmacogenomics (PGx) studies the role of inheritance of individual genetic patterns in drug response and has taken advantage of NGS technology as it provides access to high-throughput data that can, however, be difficult to manage. Machine learning (ML) has recently been used in the life sciences to discover hidden patterns from complex NGS data and to solve various PGx problems. In this review, we provide a comprehensive overview of the NGS approaches that can be employed and the different PGx studies implicating the use of NGS data. We also provide an excursus of the ML algorithms that can exert a role as fundamental strategies in the PGx field to improve personalized medicine in cancer.

## 1 Introduction

Pharmacogenetics is a branch of molecular biology and pharmacology that studies the relationships between the genetic background of individuals and the effects of a particular treatment (Nebert, 1999). In recent years, thanks to rapid access to high-throughput sequencing technologies, commonly referred to as Next-Generation Sequencing (NGS), pharmacogenetic studies have seen an upsurge in the identification of variants associated with differential patient response to drugs, leading to an evolution from pharmacogenetics to pharmacogenomics (Auwerx et al., 2022). Although these terms have subtle differences, they are generally used as synonyms and will be referred to as PGx in the following.

In many clinical trials, the primary endpoint is not met because of inadequate patient cohort selection, stratification criteria, or genotype and phenotype characterization, which in turn can introduce confounding factors and reduce the statistical power of the study (Fogel, 2018). Despite demonstrated benefit for a few patients, failure to meet the primary endpoint may reduce success rates of anticancer drugs entering clinical practice, with only about 5% of drugs approved by the Food and Drugs Administration (FDA) (Harrison, 2016). These issues are critical in cancer therapy because drugs can be ineffective for a variety of reasons, including altered expression of target genes by cancer cells and development of resistance to treatment, as well as inappropriate selection for clinical study design. As a result, many patients with advanced disease may lose access to potentially effective treatments. For these reasons, identifying the genetic factors responsible for drug response and resistance in cancer is mandatory for better patient management and treatment.

The Human Genome Project is considered a milestone in the context of sequencing experiments, and has contributed to an upsurge in both the genetic characterization of tumors and the development of sequencing technologies where NGS has become mainstream. The application of NGS technology has enabled the detection of multiple genetic mutations or altered gene expression in many samples and in a few runs, providing a large amount of data in a short turnaround time and at a competitive cost (Hussen et al., 2022). In addition, NGS allows researchers to identify somatic and germline variants within the same experiment, both of which are important in the context of cancer, drug response, and drug toxicity. Somatic variants are mutations that arise *de novo* in tissue-specific cells due to environmental stress and errors in DNA replication, and are divided into driver and passenger mutations. Driver mutations have the effect of conferring proliferative advantages, whereas passenger mutations occur in cells that already carry a driver mutation and are a consequence of genomic instability (Bozic et al., 2010). In contrast, germline mutations are inherited changes that affect reproductive cells and are present in all somatic cells; they may be common or rare in a given population. Of note, not only are somatic variants important for cancer treatment and mainly used as molecular targets, but germline mutations may also contribute, at least in part, to tumor development, progression, and resistance (Chen X et al., 2023; Wang et al., 2023).

Genetic variations in genes related to pharmacokinetic processes (PK, i.e., absorption, distribution, metabolism and excretion), or in genes related to pharmacodynamics (PD, mechanisms of action and post-target signaling), can lead to drug inefficacy or toxicity, making treatment unavailable to patients. The most commonly inherited genetic variants include single nucleotide polymorphisms (SNPs), insertions/deletions (INDELs), copy number variations (CNVs) and a variable number of tandem repeats (Ismail and Essawi, 2012; Yu et al., 2021). The frequency of these variants also plays a role in adverse drug reactions and inefficacy, as not only common variants, but also low-frequency and rare variants should be considered for drug-specific functional alterations (Lauschke et al., 2018). Processing the high-throughput data obtained by NGS in PGx approaches is challenging. To cope with this huge amount of omics data, numerous bioinformatics pipelines have been developed.

Machine learning (ML) is a branch of artificial intelligence based on statistical learning that is able to predict a response or recognize relationships between complex data structures. Thanks to its flexibility, ML is also used in medical and biological sciences (Handelman et al., 2018). So far, many efforts have been made to improve ML algorithms for cancer diagnosis, prognosis and treatment.

Existing reviews mainly address the use of omics data in cancer and pharmacogenomics, but to our knowledge very few of them focus on the use of machine learning in cancer pharmacogenomics. For these reasons, although the topics covered here are quite extensive and are not addressed in detail, this review aims to highlight emerging research trends and underlying critical issues that may be encountered by new researchers approaching ML and pharmacogenomics in cancer. In this review, we provide a comprehensive overview of NGS applications and PGx studies in personalized medicine. The first chapters are dedicated to sequencing applications (e.g., genome, exome, transcriptome), with a focus on targeted and whole sequencing approaches. We also provide an overview of omics data generated by NGS in cancer research and its application to PGx studies, focusing on targeted therapy, efficacy, and toxicity. We next analyze the types of ML algorithms and their application in cancer research. Finally, we discuss about the challenges faced by the ML approach in PGx studies and make suggestions for further improvements.

## 2 NGS approaches

**FIGURE 1:**
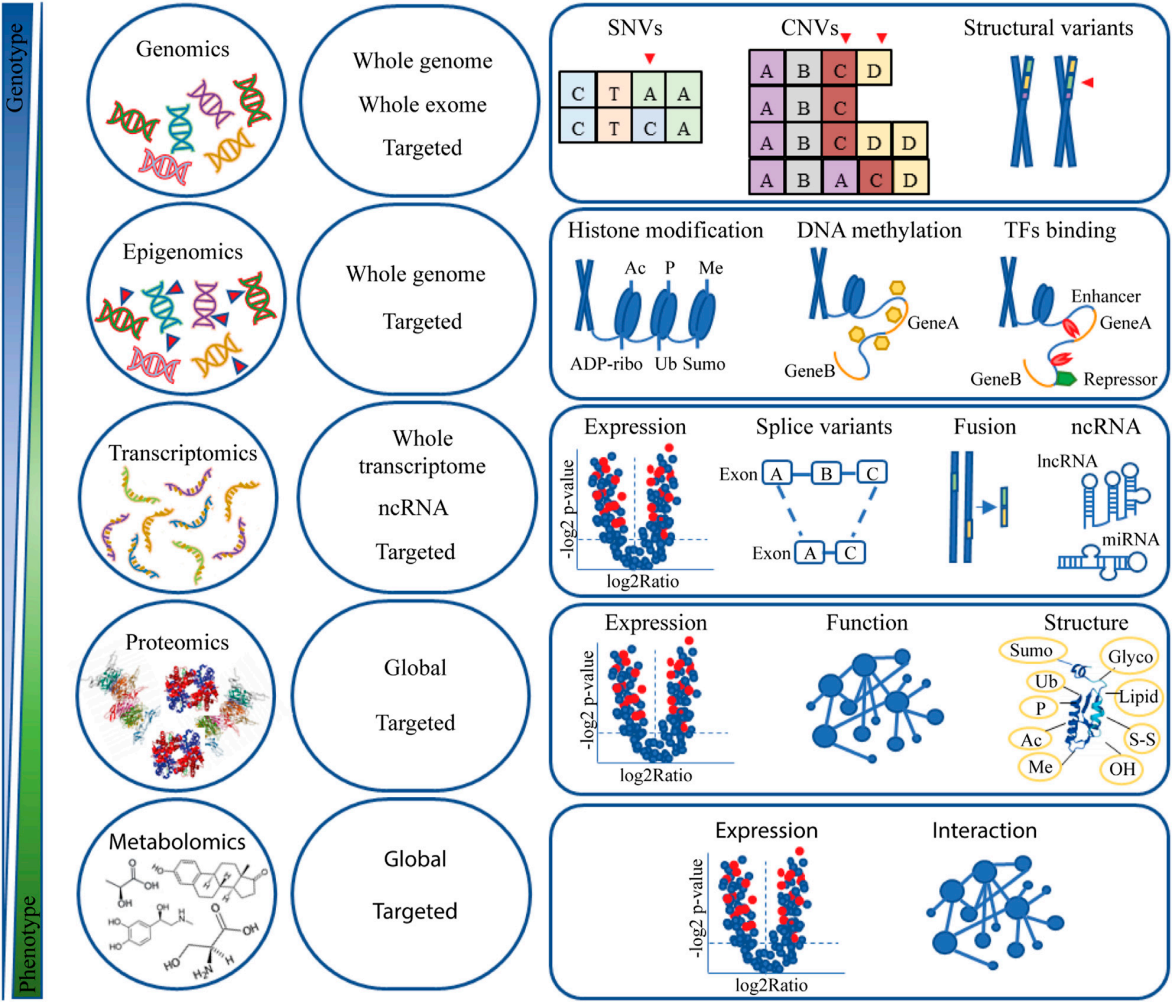
Omic data. Overview of NGS technology and applications in cancer research. Abbreviation: Ac, acetylation; ADP-ribo, ADP ribosylation; CNV, copy number variation; Glyco, glycosylation; Lipid, lipidation; lncRNA, long non-coding RNA; Me, methylation; miRNA, microRNA; ncRNA, non-coding RNA; OH, oxidrylic group; P, phosphorylation; SNV, single nucleotide variants; S-S, disulphide; Sumo, Sumoylation; TFs, transcription factors; Ub, ubiquitination.

It is widely acknowledged that NGS has been groundbreaking in cancer research. Over the past two decades, many NGS technologies have been developed to meet multiple needs and have become even more sophisticated. Figure 1 provides an overview of NGS technologies and strategies for investigating the molecular background of cancer.

### 2.1 Whole genome, whole exomes and whole transcriptome sequencing

From a theoretical standpoint, characterization of the entire genetic background of the tumor should be considered the most comprehensive strategy to gain insight into tumor biology. Whole genome sequencing (WGS) and whole exome sequencing (WES) are NGS approaches in which virtually the entire genome (WGS) or the protein-coding regions (exons) of the genome (WES) are sequenced. Both approaches can be used in cancer research to sequence normal tissue (e.g., blood) and tumor tissue to discover new targets for therapies and biomarkers of cancer stage, predisposition, and response to therapy. In addition, sequencing of paired tumor and normal tissues allows unambiguous identification of individual germline and somatic variants as well as loss of heterozygosity and the “second hit” mutations (Mandelker and Ceyhan-Birsoy, 2020).

In the clinical setting, somatic and germline variants can be identified using both WGS and WES approaches but some important aspects should be highlighted. There are two main advantages of WGS: first, the discovery of novel genomic variants, including single nucleotide variants (SNVs) and structural variants (SVs) such as CNVs, INDELs, variable stretches in tandem repeats and balanced chromosomal translocations; second, the sequencing result includes coding, non-coding and mitochondrial DNA (Sims et al., 2014). On the other hand, WES highlights coding variants that are easier to study and whose phenotypic effects are more functional to assess.

Although it could be considered an advantage to sequence the whole genome at once, since different types of variants can be found with the same sequencing library, some limitations should be considered. First, we need to distinguish between two key parameters in sequencing, coverage depth and coverage itself. Coverage depth is a measure of how often a particular base in a sequence is seen during sequencing and can be an indicator of the reliability of the results, while coverage is the percentage of the genome that is sequenced during the experiment. These parameters are closely related and must be weighed when researchers define the goal of their studies (Sims et al., 2014; Meienberg et al., 2016). Although the coverage of WGS is higher than that of WES, the average depth of coverage in WGS experiments may be low. In contrast, WES has a higher average depth of coverage compared to WGS because WES only covers the exons that account for about 2% of the genome. Another limitation is related to the data generated by sequencing, as WGS data are very huge, and processing and storing such amount of data requires adequate computational resources, which may be a limit in some contexts. Finally, the cost of WGS experiments is usually higher than WES, especially in clinical settings.

For these reasons, WES has long been considered the gold standard for detecting genetic variants. However, comparative WGS and WES studies have recently shown that WGS is more powerful than WES in exome variant detection, providing broader coverage and better variant detection, and costs are now decreasing (Belkadi et al., 2015). In addition, the latest WGS library preparation methods are PCR-free, while the WES library preparation methods still rely on PCR amplification. This could lead to GC content bias and misidentification of variants (Meienberg et al., 2016). In addition, WES does not really cover the whole exome, so some deleterious coding SNVs might be missed. As mentioned earlier, WES is not validated for the detection of structural variants, including CNVs and translocations, and finally, by definition, it does not include non-coding intron regions. Therefore, WGS has become more attractive than WES for diagnostic purposes in recent years (Belkadi et al., 2015; Lionel et al., 2018; Hou et al., 2022).

Whole transcriptome sequencing (WTS) is an RNA-based sequencing strategy that captures the transcriptome repertoire, and its applications include quantification of gene expression, detection of alternative transcripts resulting from splice variants, detection of chromosomal rearrangements leading to chimeric gene fusions, and identification of the ever-growing family of non-coding RNAs (Lakhotia et al., 2020; Li and Wang, 2021). Depending on the research interest, total RNA extracted from samples should be treated to remove unwanted RNA species, which may be a limitation, especially in terms of time. On the other hand, the loss of valuable reads and the management of background noise are problems faced when no depletion is performed. Bulked WTS has been used in cancer research to identify pathways and genes involved in cancer development and, thanks to spatial transcriptomics and single-cell sequencing, also to understand tumor organization and interactions with the microenvironment (Chen TY et al., 2023).

### 2.2 Whole vs. targeted sequencing

Many companies that have developed high-throughput technologies have now launched numerous tumor-specific panels to study cancer. Targeted panels sequence only a small part of the genome because they are designed with probes targeting specifically regions of interest, such as sets of genes, in a specific/custom fashion. Depending on the size of the panel, they can achieve the depth of coverage required to highlight specific pathogenic variants (Lenahan et al., 2023). Targeted panels offer many advantages over WGS and WES approaches, including reduced hands-on time, ease of translation of raw data, profiling of specific tumor-associated genes and customization of the panel. These advantages can support the therapeutic decision-making process while reducing the time required (Bewicke-Copley et al., 2019).

For their part, WGS and WES approaches can be extremely useful in exploratory research and clinical trials, as they do not require “*a priori*” knowledge of disease mechanisms and can reveal novel molecular biomarkers. In this sense, the COGNITION study has shown that comprehensive molecular profiling using WGS and WES identifies a genomic signature in a subset of breast cancer patients at high risk of recurrence after neoadjuvant treatment, for whom targeted therapy solutions may be available (Pixberg et al., 2022).

On the other hand, targeted panels can also be employed in clinical trial design. In this case, panels could be used for many goals. First, to stratify the cohort according to known biomarkers, as in the case of the REGISTRI phase II clinical trial, in which a customized DNA panel was developed to specifically identify *KIT/PDGFRA* wildtype GIST patients eligible for regorafenib therapy (Martin-Broto et al., 2023); second, to support the discovery of new specific positive biomarkers, associated with response to therapy, as in the RELAY phase III trial, in which a targeted approach was used to assess ctDNA mutations and *EGFR* mutation dynamics after erlotinib with or without ramucirumab treatment in NSCLC patients (Garon et al., 2023); and finally, to identify actionable tumor alterations and candidate genes for molecular targeted therapies, as demonstrated in the MATCH study (Parsons et al., 2022). Of note, many targeted panels, known as PanCancer panels, are designed to cover many cancer-related genes, so the applications of these panels are widespread for many different goals.

Whole and targeted sequencing approaches can also be combined in clinical trials, as in the EVOLVE phase II study in which WES of tumor tissue and a targeted panel of cell-free DNA from blood were matched to discover novel genomic alterations responsible for resistance to PARP inhibitors in high-grade serous ovarian cancer (Lheureux et al., 2023).

In the clinical setting, things are different, as the cost of analysis is one of the limiting factors for sequencing. In this scenario, targeted panels are preferred because they have a lower cost per sample and an easier data management (Bewicke-Copley et al., 2019). Targeted panels are often designed to provide information on known biomarkers such as genomic instability score (GIS), loss of heterozygosity (LOH), microsatellite instability (MSI), and tumor mutation burden (TMB). The latter biomarker is of great interest in clinical practice, as pembrolizumab is the first FDA-approved agnostic cancer therapy that can be used in tumors with high TMB (Marcus et al., 2021); however, the sequencing method used to assess TMB may impact clinical outcomes, by excluding patients who might otherwise benefit from this treatment. Indeed, WGS, WES, and targeted-based panels have been used to measure TMB in cancer patients, with not only primary tumors but also circulating DNA in blood proposed as an alternative source material. In this context, WES of tumor and paired normal tissues is currently considered the most accurate approach to determine TMB, although this approach is both costly and time-consuming in the clinical setting (McGrail et al., 2021). On the other hand, an approach that uses targeted sequencing assays enriched in genes known to be involved in cancer appears to be more feasible in the clinic, particularly because these assays do not require paired tumor and normal samples to determine TMB. However, the type of cancer tested and the type of panel used to assess TMB can significantly affect the outcome (Merino et al., 2020). Among the many examples of targeted panels used in the clinical setting there are MyeloSeq, a 40-gene targeted panel used to determine variant and allele frequencies in patients with suspected hematologic malignancies (Barnell et al., 2021), the Oncomine Precision Assay, which tests 45 cancer-related genes (such as *EGFR, KRAS, ALK, RET, BRAF,* and others) used in screening for genomic alterations that can be treated with targeted therapy in NSCLC, colorectal cancer, melanoma, breast cancer, and other malignancies (Werner et al., 2022; Nindra et al., 2023), and other targeted panels such as the TruSight Oncology, the AmpliSeq, the FusionPlex, the QIASeq Multimodal Lung, which have demonstrated expertise in identifying genetic variants, and the *NTRK* gene fusion panel used to identify tumors sensitive to larotrectinib, an NTRK inhibitor (Drilon et al., 2018; Stockley et al., 2023).

In addition, there is also an ethical aspect to consider, as sequencing a larger portion of the genome may lead to the identification of unsolicited findings that should be better communicated to physicians and patients (Schoot et al., 2021). Targeted panels therefore have a lower chance of discovering new unsolicited findings, which facilitates clinical reports.

In summary, although WGS and WES sequencing are accurate and do not require “*a priori*” knowledge of disease mechanisms, their introduction into clinical practice may not be feasible, mainly because of coping with the volume of data and the cost per sample. On the other hand, targeted sequencing may facilitate the introduction of bulk sequencing into routine clinical practice where testing of multiple molecular biomarkers has become common practice, as has been the case with TMB and NTRK gene fusions. Again, the best choice between the two strategies must be balanced between cost and application.

### 2.3 Epigenomics

Epigenomic sequencing has proliferated in recent years. Epigenetics is a branch of biology that studies the causal interactions between genes and their products. Basically, epigenetics studies all changes and phenotypes in gene expression that cannot be attributed to genetic causes. The most important changes can occur directly at the DNA, e.g., cytosine methylation, or at the chromatin proteins, e.g., acetylation, methylation, phosphorylation, and others (Kouzarides, 2007). The consequence of these changes is the modulation of the accessibility of the DNA sequence to enzymatic complexes, which determines the state of gene activation.

Sequencing of epigenetic modifications (epigenomics) identifies specific cancer signatures involved in tumorigenesis as well as cancer metastasis and recurrence (Huang et al., 2018; Malta et al., 2018; Sengupta et al., 2021). In particular, some histone modifications, such as reduced lysine acetylation and methylation, may act as prognostic biomarkers in breast cancer (Elsheikh et al., 2009; Zhou et al., 2022) or they may predict response to treatment, as in the case of immunotherapy (Peng et al., 2015; Hoffmann et al., 2023). In addition, dysregulation of genes involved in chromatin remodeling can also be a hallmark of a particular tumor. This is the case with mutations of histone deacetylase (HDAC) in multiple myeloma and lymphoma. HDAC inhibitors and DNA methylation inhibitors are anticancer drugs developed to target the aberrant activity of these molecules (Blumenschein et al., 2008; Galanis et al., 2009).

### 2.4 Proteomics and metabolomics

Proteomics and metabolomics are high-throughput screening of protein expression and metabolite abundance, respectively. Proteomics data can be considered a readout of the transcriptome, but it has been reported that only 40% of protein expression can be explained by a corresponding gene expression profile (Ideker et al., 2001; Vogel et al., 2010). Proteomic studies take pictures of the cellular protein repertoire that include protein abundance and turnover, post-translational modifications, subcellular localization, interactions with other proteins and structures, and finally protein involvement in metabolic pathways (Altelaar et al., 2013).

On the other hand, metabolomics studies investigate the presence of metabolites, their concentration and their interactions with biological systems. Unlike other “omics” approaches, metabolomics can reflect the actual biochemical activity and the state of cells and determine the true cellular phenotype (Patti et al., 2012; Tan et al., 2012).

In cancer research, proteomics and metabolomics strategies have been used not only to identify novel biomarkers involved in tumor resistance and signature that predicts treatment outcome (Dytfeld et al., 2016; Shrestha et al., 2021; Robles et al., 2022), but also to uncover cancer metabolic pathways and oncometabolites that may drive tumorigenesis and sustain tumor progression (Xu et al., 2011; Drusian et al., 2018).

## 3 Omics data in cancer personalized therapy

Over the past 20 years, molecular assessment of tumors has entered routine clinical practice and has been incorporated into the WHO classification criteria for tumor diagnosis, grading and prognosis (Organisation mondiale de la santé and Centre international de recherche sur le cancer, 2020; Sbaraglia et al., 2020; Louis et al., 2021).

As a result, the treatment of patients shifted mainly towards tailored therapies, and the development of new classes of anticancer drugs increased, defining the beginning of the molecular era of targeted therapy. The so-called “targeted therapy” refers to drugs that are aimed at interfere with specific molecular target that is thought to play an important role in tumor development and progression. One of the first and most successful examples of targeted therapy is imatinib, a small molecule receptor tyrosine kinase (RTK) inhibitor that targets a variety of RTKs. The use of imatinib in tumors harboring activating mutations of RTKs (e.g., gastrointestinal stromal tumors, dermatofibrosarcoma protuberans) or oncogenic RTK fusion proteins (e.g., chronic myeloid leukemia positive for the *BCR::ABL* fusion, myelodysplastic/myeloproliferative disorders associated with *PDGFR* gene rearrangements), leads to increased life expectancy for patients whose prognosis was previously very poor (Druker et al., 1996; Druker et al., 2001; Demetri et al., 2002).

The specificity of targeted therapies usually gives these drugs particularly high efficacy while reducing off-target toxicity to normal cells, but it is also responsible for mechanisms of tumor resistance. It is noteworthy that not only the presence of a mutated drug-responsive gene, but also the type of mutation on the same gene can play a role in drug response. The *EGFR* gene in particular can serve as an example. Most glioma tumors are dependent on EGFR signaling, making approved drugs targeting this gene attractive for precision oncology of gliomas. However, most clinical trials have failed to demonstrate the benefit of EGFR-targeted therapies in gliomas, as approved EGFR therapies have mainly focused on NSCLC *EGFR* gene alterations that are, however, distinct from those driving gliomagenesis (Lin et al., 2022). On the other hand, secondary resistance occurs when a fraction of cancer cells develops new acquired mutations or alterations in antigen presentation, which can also drastically affect the efficacy of targeted therapies. Transcriptional deregulation and de-repression of alternative RTK are also common strategies to facilitate adaptive evasion signaling, which is likely promoted by epigenetic changes (Jun et al., 2012; Akhavan et al., 2013). The presence of resistant cells forces clinicians to dose escalate with the risk of increased toxicity or to switch molecular targets with the risk of no new therapeutic options being available.

In the scenario of personalized cancer therapy, the assessment of the individual status of the tumor immune system also plays an important role and has led to the development of nanoparticles, monoclonal antibodies, and chimeric antigen receptor T-cell therapies (CAR-T) as well as antibody-drug conjugates. Some examples include the generation of third-generation CAR-T cells against the oncoembryonic antigen ROR1 (Meng et al., 2023) and the study of the chemokine expression signature that correlates with the characteristics of T-cell inflammation and potential response to immune checkpoint inhibitors in various cancers (Romero et al., 2023).

Another goal of precision medicine is to identify new molecular biomarkers that can monitor both disease stage and the efficacy of selective therapies. In this context, tumor molecules secreted in the bloodstream, such as microRNAs, non-coding RNAs, exosomes, and circulating tumor DNAs (ctDNAs) are of particular interest and can be detected without invasive procedures thanks to liquid biopsy. For example, the expression of circulating miR-221/222 correlates with response to and resistance to tamoxifen in the luminal subtype of breast cancer patients (Patellongi et al., 2023), circRNA_047733 can be used as a biomarker for risk assessment of lymph node metastasis in patients with oral squamous cell carcinoma (Deng et al., 2023), and baseline ctDNA mutation frequency can be used as a prognostic marker in patients with metastatic colorectal cancer (Bachet et al., 2023). In addition, measurement of ctDNA at specific time points by liquid biopsy can also be used as a biomarker of efficacy and toxicity to guide the dose and schedule of radiotherapy in cancer patients (McLaren and Aitman, 2023).

Finally, several cancer hospitals have interdisciplinary teams of experts, called molecular tumor boards, that recommend a patient-specific therapeutic strategy based on data from NGS profiling. A retrospective study of biliary tract tumor patients showed that comprehensive genomic profiling along with molecular targeted therapy discussed by the molecular tumor board resulted in a higher response rate and better overall survival for patients who received the recommended treatment (Zhang D et al., 2023). In addition, these molecular tumor boards can bridge the gap between research and the clinic by recruiting patients early for clinical trials (Weiss et al., 2023).

As discussed in the following section, various genetic polymorphisms that can be studied using omics experiments and that are located in genes associated with drugs PK and/or PD, can influence efficacy and toxicity.

### 3.1 Omics data in PGx studies

**FIGURE 2:**
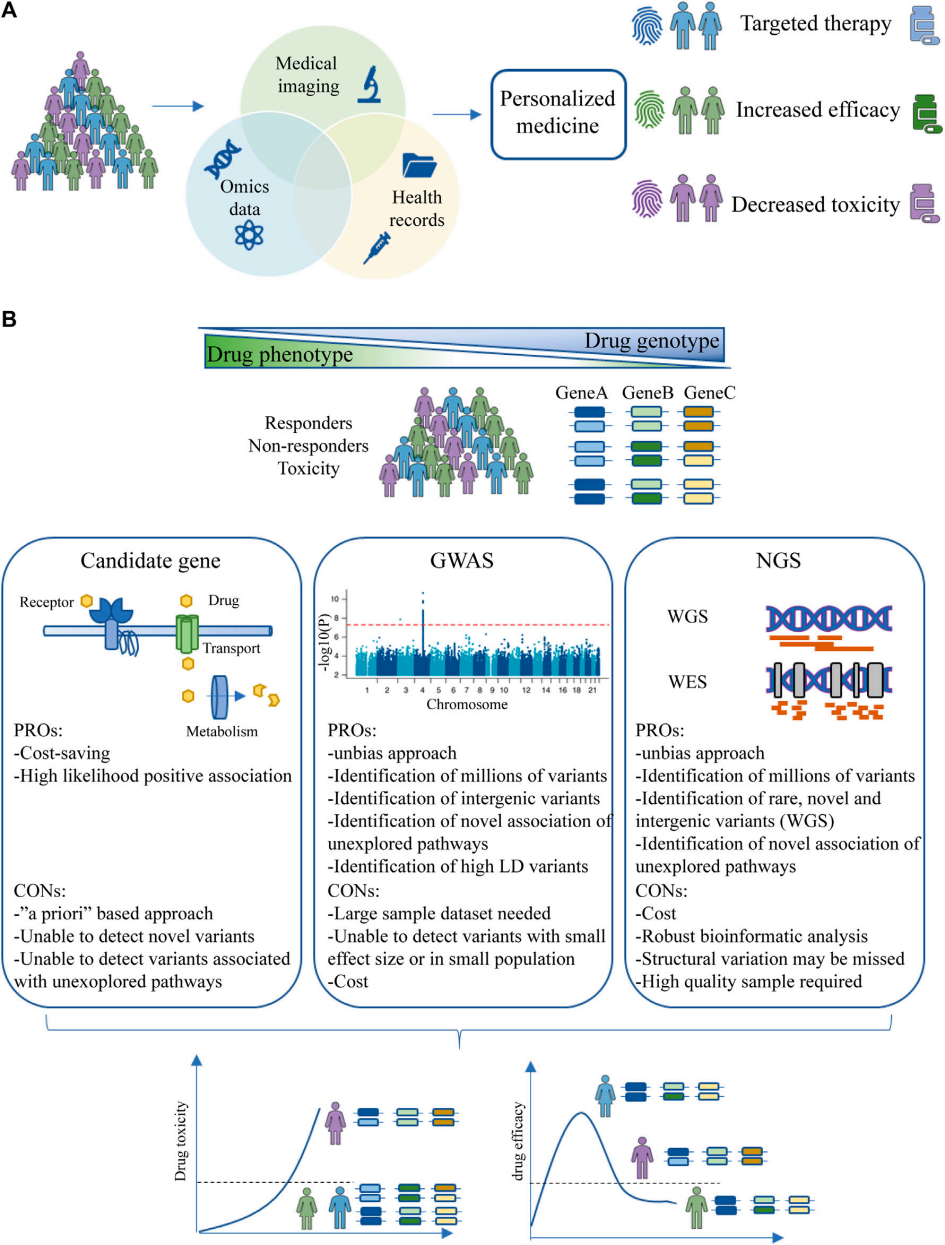
Personalized medicine and PGx studies. **(A)** Aims of personalized medicine. Patient data from multiple sources (health records, medical imaging and omics data) are combined to identify a patient-specific fingerprint that determines response to therapy, efficacy and toxicity. **(B)** PGx study design strategy. Three drug phenotypes will be identified (responders, non-responders and toxicity) and different populations will be studied to identify the genetic traits involved in particular drug phenotype. The strategies are divided into candidate genes, GWAS and NGS. The aim of PGx studies is to stratify the population based on genetic background to maximize drug efficacy and reduce toxicity. Abbreviations: GWAS, Genome-wide Association Study; NGS, Next-Generation Sequencing.

In addition to targeted therapy, NGS approaches can support the concept of personalized medicine by being included in PGx studies and linked to drug safety profiles (Figure 2A). Sequencing results can thus be associated with the identification of novel biomarkers related to drug efficacy and toxicity.

The three main research strategies for PGx biomarker discovery are candidate gene studies, Genome-Wide Associated Studies (GWAS), and NGS (Figure 2B). Candidate gene studies are based on genotyping or sequencing of genes known to be involved in PK and PD processes to uncover potential variants; this is the main approach taken so far. This approach is based on “*a priori*” knowledge, as genes are selected based on their membership in specific pathways (Malta et al., 2018; Kwok et al., 2022; Maeda et al., 2023). A limitation of this approach is that polymorphisms that are part of unexplored pathways and may alter the phenotype of the drug response are not detected. However, this approach may have higher statistical power than other approaches even with few samples (Chan et al., 2019).

On the contrary, GWAS discovers millions of SNPs across the genome and has the potential to find variants in unexplored genes and intergenic regions not previously thought to affect drug response (Uffelmann et al., 2021). One example is the germline variants in the *PRUNE2* and *BARD1* genes, which have prognostic potential in advanced colorectal cancer and ovarian cancer, respectively. In addition, the variants in the *AGAP1* gene may affect patient response to bevacizumab (Quintanilha et al., 2022b). Moreover, most of the SNPs detected are not the causal variants responsible for the observed phenotype, but are instead associated with the presence of functional variants in high linkage disequilibrium in a given population (Bei et al., 2010). One of the major limitations of this technique is the low statistical power in detecting associated signals for rare polymorphisms and thus the inability to find variants with small effect sizes, especially when a drug effect trait is not directly associated with drug effect or is population/region specific (Wang et al., 2019).

Finally, NGS approaches offer the possibility of generating a large amount of information on novel, common, or rare variants potentially associated with drug response, such as GWAS, but suffer less from the requirement of a large number of samples to achieve statistical power, overcoming the drawbacks of the other two SNP approaches (Sharma et al., 2014; Auwerx et al., 2022). However, NGS is not a panacea for identifying all inheritance patterns in PGx. The lack of standardization of NGS techniques and limitations in sample quality or quantity are issues that should be addressed to achieve comprehensive and robust detection and association of somatic and germline variants involved in individual drug response (Mu et al., 2019).

Although candidate gene studies, GWAS, and NGS enable the identification of variants potentially involved in individual drug response, the results obtained in these studies require internal and external validation before they can be adopted in clinical practice. Validation strategies include case/control studies, cross-validation based methods, and an independent series of patients to confirm results. In addition, orthogonal technical validation using low-throughput methods is required, with real-time PCR with TaqMan assay and pyrosequencing often being the first choice (Arbitrio et al., 2021; Hertz et al., 2021).

#### 3.1.1 PGx in drug response: focus on efficacy

The efficacy of a drug is related to its plasmatic concentration, which is a surrogate for measuring the percentage of the administered dose that can reach the molecular target. Genetic variants in genes involved in PK or/and PD can affect the efficacy of a drug, as discussed below. Although much of the variation is due to PK (Roden et al., 2019), PD variations may also be important for treatment efficacy.

Classic examples of PGx in drug response involving PK mechanisms include glutathione S-transferase, cytochrome *P450* genes and *MDR1* gene polymorphisms. Glutathione S-transferase (GST) is a class of metabolic enzymes that conjugates glutathione to xenobiotics for detoxification purposes. GST substrates include anthracyclines and cyclophosphamide, anticancer drugs used in breast cancer protocols. In particular, the GG genetic variant in the *GSTP1* gene (c.313A>G) appears to be associated with a lower risk of chemoresistance in breast cancer patients treated with doxorubicin (Romero et al., 2012) and a lower risk of death in patients treated with cyclophosphamide compared to patients with proficient-GST (Sweeney et al., 2003). One possible mechanism to explain these observations is that decreased GST activity leads to an increase in the systemic dose of active metabolites, which in turn results in a better therapeutic effect, even if this increases the risk of toxicity.

Cytochrome P450 is one of the most important enzyme classes involved in the metabolism of xenobiotics. This group includes the enzyme CYP2D6, which is involved in the conversion of tamoxifen to its more active metabolites, 4-hydroxytamoxifen and endoxifen. Genetic variants in the *CYP2D6* gene (*4, *5, *10 and *41) result in impaired enzyme activity, leading to lower production of active tamoxifen metabolites and shorter overall survival in cancer patients taking tamoxifen (Schroth et al., 2007).

Genetic variants affecting transporters may also play a role in altered drugs response. A silent polymorphism in the *MDR1* gene, one of the best-known efflux pump proteins involved in drug resistance mechanisms, has been shown to affect the timing of MDR1 mRNA translation into folded protein, thereby reducing total protein levels (Kimchi-Sarfaty et al., 2007).

Novel PK-related genetic variants have also been discovered. Germline polymorphisms in the *NT5C2* gene (e.g., rs72846714) were recently discovered in a GWAS study, and some of them have been linked to 6-mercaptopurine (6-MP) metabolism in patients with acute myeloid leukemia, as they are responsible for differential activation of 6-MP and thus its bioavailability (Jiang et al., 2021).

At PD, it can be speculated that any variant (germline or somatic) that affects the accessibility of the drug to its target or the affinity of the drug binding may result in altered drug efficacy. These scenarios include variants that alter the amino acid sequence at the core binding site between the drug and the target, thereby affecting binding affinity, variants that alter the spatial conformation of the protein and may lead to partial misfolding, and alterations in weakly bound bridges between individual nucleic bases. In NSCLC patients treated with the EGFR inhibitor gefitinib, patients with specific in-frame indel mutations in the *EGFR* gene were more sensitive to gefitinib, as these mutations increase the tumor’s dependence on growth factor signaling, compared to patients without such mutations. Therefore, patients with these mutations respond better to gefitinib than patients who have other mutations (Lynch et al., 2004).

In glioblastoma, *EGFR* mutations and amplifications account for at least 50% of molecular alterations (Brennan et al., 2013). The EGFR variant III (EGFRvIII) is the product of the most common deletion in GBM, resulting from the deletion of exon 2-7 of the extracellular domain (ECD). This alteration occurs predominantly in cancer cells and in approximately one-third of GBM, making this variant an ideal epitope for immunotherapy. Rindopepimut is a peptide-based cancer vaccine that targets EGFRvIII. Although EGFRvIII is an extremely attractive therapeutic target, tumor cells escape this immune-mediated therapy by losing the EGFRvIII expression as a resistance mechanism (Binder et al., 2018).

Epigenetic changes may also affect PGx. Hypermethylation of *MLH1*, which is involved in the mismatch repair system, may affect the response to cancer therapy targeting this pathway (Wu F et al., 2015; Bukowski et al., 2020; Loukovaara et al., 2021). In addition, altered histone modifications that may occur during tumorigenesis and other pathological conditions may lead to heterogeneous expression of drug efflux proteins and thus affect PK (Kondo and Issa, 2004; Baker et al., 2005; Wu L-X et al., 2015). In particular, demethylation of the *ABCB1* gene in cancer cells can lead to a reduction in the accumulation of anticancer drugs in cancer cells, resulting in the acquisition of a resistant phenotype (Toth et al., 2012).

#### 3.1.2 PGx in drug response: focus on toxicity

Drug toxicity refers to a variety of adverse effects associated with the use of a particular drug. The mechanisms of drug toxicity can vary widely and include four main aspects: on-target toxicity, off-target toxicity, hypersensitivity reactions and idiosyncratic reactions (Guengerich, 2011). Genetic polymorphisms may enhance or attenuate these reactions.

On-target toxicity refers to the adverse effect of a particular drug depending on its mechanism of action. This phenomenon is related to the binding of the drug to its therapeutic receptor, but in a different body compartment. Polymorphisms that enhance this type of response include rs9501929 of the *TUBB2A* gene, which encodes the β-tubulin protein. Although there are conflicting opinions about its clinical utility, rs9501929 may alter the toxicity profile of paclitaxel, an antimitotic drug that binds specifically to β-tubulin to arrest cell cycle progression. Patients carrying this variant have a higher risk of developing paclitaxel-induced neuropathy, a disease characterized by abnormal aggregation of microtubules in neurons (Abraham et al., 2014). This effect can be explained by the fact that β-tubulin is also targeted by paclitaxel in normal neurons, which is, by definition, an on-target toxicity. The lack of a selective and specific target for cancer cells is one of the major limitations of conventional chemotherapy such as paclitaxel.

Off-target toxicity refers to the adverse effects of a drug that binds to both its therapeutic and nontherapeutic receptors and is also related to mechanisms that are independent of the mechanisms of action (Rudmann, 2013). Some of these issues can be addressed in the preclinical stages of drug development by altering the structure of the drug to modulate its affinity to undesirable receptors. In addition, local administration, if applicable, may also partially help (Li M et al., 2022). Hypersensitivity reactions and idiosyncratic reactions depend on the activation of the immune system and the intrinsic characteristics of patients, respectively (Doña et al., 2014).

The best characterization of adverse reactions involves genes from PK processes. Individual variants in transporters and metabolic enzymes are responsible for most differences in drug response. In particular, polymorphisms in the gene *SLCO1B,* which encodes the OATP1B1 transporter responsible for cellular uptake of multiple substrates, can impair the availability of irinotecan and lead to drug toxicity (Nozawa et al., 2005; Di Martino et al., 2011). In addition, part of irinotecan is oxidized by CYP3A4, while another part of irinotecan is activated to SN-38 and its glucuronide conjugate SN-38G. These reactions are catalyzed by carboxylesterase and UGT1A1, respectively. The genetic variant UGT1A1*28 is associated with decreased glucuronidation activity, which in turn prolongs the mean half-life of the metabolite SN-38 and increases patient susceptibility to gastrointestinal and hematologic toxicity (Iyer et al., 2002; Innocenti et al., 2004; Peeters et al., 2023). Similar toxicities to irinotecan have been noted in patients carrying polymorphisms in the *ETS1* and *ABCG2* genes, which encode carboxylesterase and a membrane transporter, respectively (De With et al., 2023). The UGT1A1*28 polymorphism, together with the UGT1A1*60, UGT1A1*6, and UGT1A1*27 polymorphisms is associated with the metabolism of several anticancer drugs such as belinostat, an HDAC inhibitor, nilotinib and pazopanib, two RTK inhibitors. Thus, loss-of-function alleles are responsible for increased toxicities, such as neutropenia, thrombocytopenia and prolonged QTc intervals in patients treated with belinostat (Goey et al., 2016; Balasubramaniam et al., 2018).

Another example is 5-fluorouracil (5-FU), an antimetabolite that has long been used to treat tumors of the stomach, colon and rectum. Approximately 80% of 5-FU is converted to the inactive metabolite 5,6-dihydrofluorouracil by the rate-limiting enzyme DihydroPYrimidine Dehydrogenase (DPYD). Genetic mutations in the *DPYD* gene associated with lower DPYD activity, such as *2A, *13 and rs67376798, can lead to fluoropyrimidine toxicity (Caudle et al., 2013; Glewis et al., 2023; Lešnjaković et al., 2023).

Germline mutations in the *TPMT* gene, as well as in the *NUDT15* gene, may otherwise affect the metabolism of the thiopurines 6-mercaptopurine (6-MP) and 6-thioguanine (6-TG). In thiopurine metabolism, TPMT is a key enzyme that converts 6-MP and 6-TG to their inactive metabolites. Patients with loss of function alleles have higher circulating thiopurines levels, which increases the risk for developing myelosuppression, a common adverse effect of these drugs. In these patients, a lower starting dose is recommended to minimize toxicities (Wang et al., 2010). The same precautions should be observed in patients with loss-of-function alleles of the gene *NUDT15*, which is also involved in the metabolism of thiopurines (Moriyama et al., 2016). Genetic testing for *TPMT* and *NUDT15* genes has entered clinical practice and is strongly recommended for cancer patients who are to receive thiopurine therapeutics (Relling et al., 2019).

Some of the classic gene polymorphisms such as cytochrome *P450, DPYD, UGT, TPMT*, and *HLA* have already entered clinical practice. Panels of specific genes are routinely used to determine the optimal therapeutic window in cancer patients and their utility has been demonstrated. A recent multicenter implementation study evaluated a panel of 12 genes for pharmacogenetic testing in several European countries. The most important finding of this study, in addition to demonstrating that genotyping of this 12-gene panel leads to a reduction in the incidence of relevant adverse drug reactions, is the cross-national feasibility of these genetic tests, which paves the way for harmonization of genotyping (Swen et al., 2023).

In summary, PGx testing offers a number of benefits, including enhancing intended treatment benefits, reducing the likelihood of adverse effects and risk of dependence, reducing healthcare expenditures and the need for hospitalization in the event of severe adverse events, and shortening the time to achieve therapeutic effect. Although researchers and clinicians are increasingly aware of the importance of genetic testing for personalized oncology, global clinical implementation is still lacking, in part due to the need for standard procedures, cost reduction, but also support from healthcare systems, especially in less affluent countries.

## 4 Machine learning in cancer research

Machine learning (ML) is a subfield of artificial intelligence that aims to make predictions and inferences within a certain range of accuracy by analyzing multiple variables in input data, such as clinical and/or molecular data (Mitchell, 2013). In addition, without explicitly programming, ML can find hidden patterns and identify relationships between multiple variables to correctly predict the outcome.

Machine learning has indeed proven to be a powerful tool in cancer research, as it has the potential to improve cancer diagnosis, classification and prognosis (Kourou et al., 2015; Cui et al., 2022a). An example of the use of ML in cancer research is the study of medical imaging, where large amounts of data are available that are difficult to analyze. In particular, the emerging field of radiomics uses images routinely produced in clinical settings to evaluate patients undergoing treatment to develop a ML approach to disease detection (Lambin et al., 2012). Another example is radiogenomics, where key features extracted from radiological images can be linked to the genetic profile of the tumor. Thanks to the linkage of image and genotype highlighted by ML, radiogenomics offers the possibility of becoming a noninvasive surrogate for genetic testing (Meißner et al., 2022).

Other important approaches of ML in cancer research focus on treatment. Predicting how a particular tumor will respond to therapy, or which patient characteristics better predict response to therapy, is a fundamental goal of modern oncology that should ultimately lead to tailored treatment. For example, genetic profiles and clinical information of breast cancer patients from a complete study dataset were used to train a ML algorithm to predict the 5-year survival rate of these patients who underwent a specific medication (Tabl et al., 2019). In this context, genomic profiling can provide information about the role of biological pathways in cancer cells and their relationship with a specific medication, thus helping clinicians to tailor treatment for patients based on their molecular background.

### 4.1 Machine learning at a glance

A detailed description of ML is beyond the scope of this review; however, we provide here an overview of the main features of the algorithms of ML.

**FIGURE 3:**
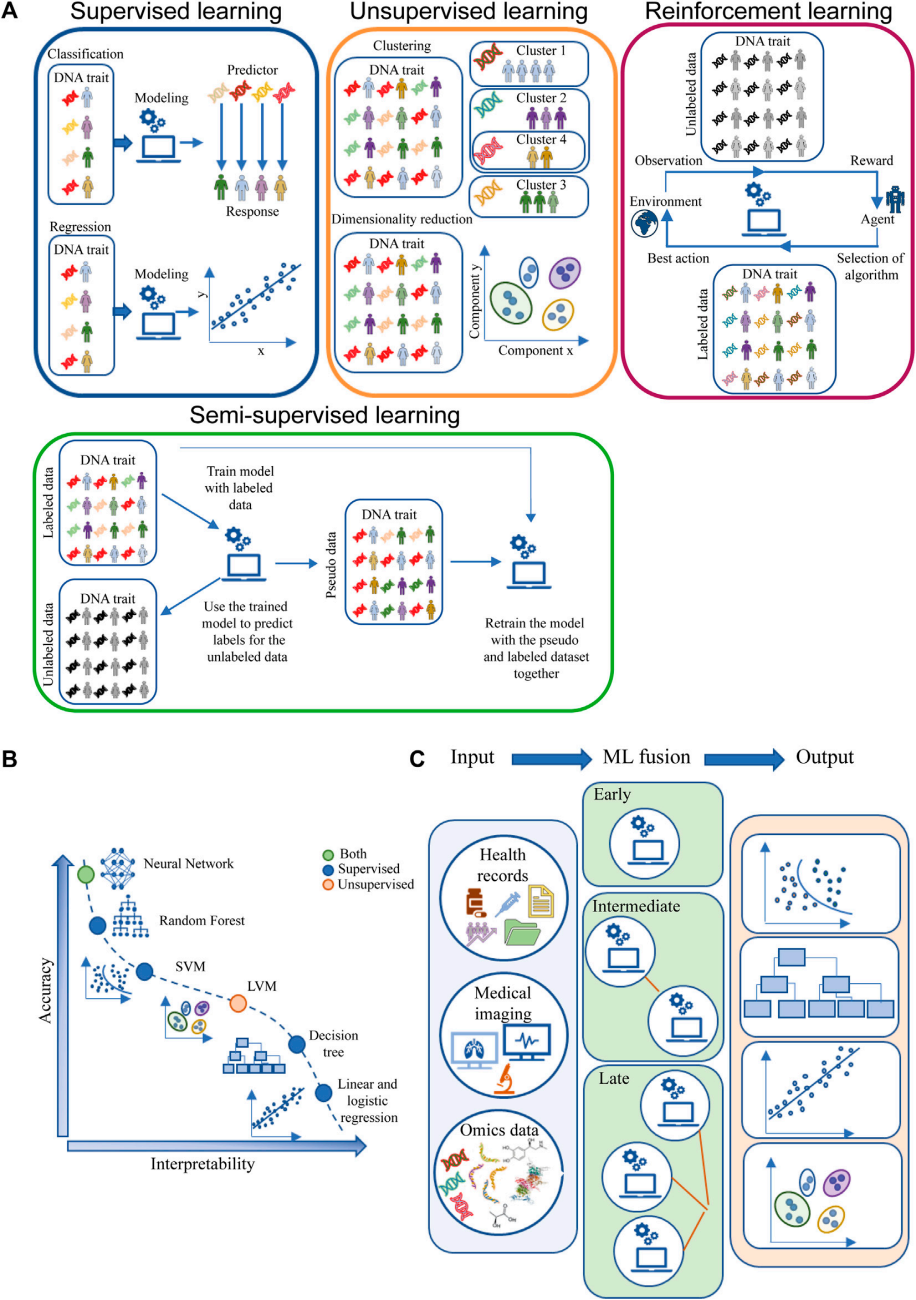
Machine learning algorithms. **(A)** ML main classification based on input data labels and possible outputs. **(B)** Trade-off between accuracy and interpretability for ML models grouped by supervised, unsupervised or both types of algorithms **(C)** ML fusion modeling. The light blue box represents the input data (health records, medical imaging and omics data), the green box represents the type of ML fusion: early fusion (top) computes a single ML model; intermediate ML fusion (middle) computes two or more ML models, with the final model using the output of previous model as input; late fusion (bottom) creates multiple ML models and then fuses the outputs of each model to produce the data output. The light red box represents examples of the outputs obtained through ML, from the top to the bottom: Classification, Decision Tree, Regression, Clustering. Abbreviation: LVM, latent variable model; SVM, support vector machine.

Supervised learning, unsupervised learning and reinforcement learning are three main types of machine learning approaches (Van Der Lee and Swen, 2023). A more classical classification based on the model built using this approach is divided into supervised, unsupervised and semi-supervised models based on the type of input data, i.e., whether it is labeled, unlabeled or a combination of both (Koteluk et al., 2021; Naik et al., 2023). A graphical representation of the concept is shown in Figure 3A.

In supervised models, which account for the majority of published ML methods, each data point contains an associated label (correct/expected response) for which a ML model must be developed. Typically, the data is split into two subsets: the training data and the testing data. The training data is used to tune and train the model (Shahin et al., 2023). The testing data is used to evaluate the generality of the model. In addition, supervised learning can be used to solve regression and classification problems. In regression problems, the labels are continuous values, while in classification problems labels are discrete values. Finally, the metrics used to assess the quality of the model depend on the nature of the problem and the type of application (Boyd, 2010; Sra et al., 2012; Orozco-Arias et al., 2020; Woodman and Mangoni, 2023).

In unsupervised models, the output label measurement is usually not available. In this case, the algorithm learns relationships from data structures to provide latent patterns that need to be evaluated for utility. However, this process still requires human intervention to validate the output variable. In general, unsupervised methods deal with clustering and dimensionality reduction, leading to the identification of subgroups with common features, which is one of the main applications of unsupervised ML (Sajda, 2006; Handelman et al., 2018).

Reinforcement learning is a type of learning where an agent learns to make decisions by interacting with an environment. The agent receives feedback in the form of rewards or penalties for its actions and learns to maximize the cumulative reward over time (Woodman and Mangoni, 2023). Unlike supervised and unsupervised learning, reinforcement learning does not require labeled data or a training set. This type of learning is often compared to a scenario where an agent learns through trial and error (Shahin et al., 2023). Reinforcement learning can be used to solve problems that deal with complex dynamics that are influenced by changing stimuli and conditions, such as in the real clinical world (Eckardt et al., 2021; Ryan et al., 2023). In particular, reinforcement learning could help physicians to select the right therapeutic regimens for a patient, and it is able to correct its predictions based on the observation of the adverse reaction resulting from the interaction between the agent and the environment (Niraula et al., 2021; Ryan et al., 2023).

Semi-supervised approach is a method in which there is a mixture of labeled and unlabeled data (Ge et al., 2020; Shi et al., 2021; Roy et al., 2022). There have been significant developments in the field of semi-supervised learning, as researchers have proposed various techniques to make effective use of the combination of labeled and unlabeled data. These techniques aim to overcome the challenges posed by the limited amount of labeled data and the growing volume of unlabeled data (Eckardt et al., 2022; Dou et al., 2023).

In ML it is important to note the differences between prediction and inference. These two terms, often used as synonyms, are used differently in ML algorithms. Prediction is about estimating or predicting unknown outcomes, while inference is about understanding the factors and relationships that contribute to those predictions. Both aspects are crucial in ML, as prediction enables useful forecasts, while inference helps to gain insights into the underlying mechanisms and to learn from the trained models (James et al., 2013). Depending on the objective, ML algorithms have been developed to make predictions, inferences or a combination of both.

An important issue to address when discussing ML is the trade-off between model accuracy and interpretability. Some approaches are easier to interpret, but are more rigid and less accurate, because they may be based on linear functions such as linear regression. Conversely, other ML models are more flexible in estimating the functional form of the function but can be difficult to explain (Eckardt et al., 2022; Kang et al., 2023). Figure 3B illustrates the trade-off between flexibility and interpretability for some of the most commonly used ML approaches.

ML input data can be of different origins, e.g., clinical data, medical imaging, omics, time series. The use of a single type of input data is characteristic of unimodal ML, while the use of different types of input data is a feature of multimodal ML. Each type of data can be modeled in different ways, resulting in early, intermediate and late fusion (Figure 3C). In early fusion, the input data types are merged at the beginning to create a single ML model. In intermediate fusion, ML models are created interlocked, each refining the previous model. Late fusion creates separate unimodal models that are combined into a final model. The multimodal ML provides more comprehensive and accurate predictions than unimodal models. Moreover, within the multimodal approaches, the intermediate and late fusion strategies achieve better results because they take complementarity information into account when training the model (Steinberg et al., 1999; Kline et al., 2022). In cancer research these multimodal approaches are considered very useful, but their application is not so obvious. ML late fusion strategies can be used to improve the oldest diagnosis criteria, tumor classification and subtype identification, as in the case of NSCLC, where a study shows that fusion of 5 different sources of information achieves the better performance in classification compared to algorithms using only single source information (Carrillo-Perez et al., 2022). In addition, they can be used to develop software for cancer theranostics, a cancer control strategy that combines early diagnosis, accurate molecular imaging, and personalized radiation treatment (in terms of chosen agent, dose, and timing) based on the individual omics profile.

### 4.2 Machine learning algorithms and deep learning applications

**FIGURE 4:**
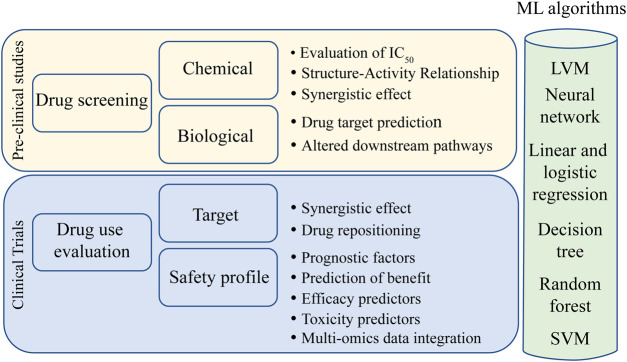
Main uses of ML algorithms in cancer research. Light yellow box represents preclinical studies where ML has been demonstrated to be effective. Light blue box represents the phases of drug utilization in target populations where ML has taken improvements. In the green light column, different ML algorithms that can be used for each research topic. Abbreviations: LVM, latent variable model; SVM, support vector machine.

Numerous ML methods have been developed for medical research and recent applications of ML are summarized in Table 1. Here we give an overview of the main algorithms used in the field of oncology (Figure 4), namely, k-means clustering and hierarchical clustering, latent variable model, support vector machine, decision tree learning, and neural networks. For neural network algorithms, we focus on Deep Learning (DL), which is becoming increasingly important in cancer research.

**TABLE 1:** ML algorithms in cancer research in the last 2 year. In this table, we summarize the most important research topics in cancer research using ML. For each publication, we describe the type of the ML algorithm used and the research outcome of the selected study. Publication years: 2021–2023.

Research topic	ML algorithm	Outcome	References
IC_50_ value	NN	*In silico* model that estimates IC_50_ values	Ma et al. (2022)
Structure-activity relationship	Decision tree	Analysis of SAR of HDAC1 inhibitors	Li et al. (2023)
Drug target prediction	SVM	Ligand- and structure-based identification of novel CDK9 inhibitors	Zhang et al. (2022)
Synergistic effect	RF	Synergistic drug combinations in CRC tumors using metabolomic data	Lv et al. (2022)
Pathway alteration	SVM	Identification of biological pathways involved in cancer drug response	Zhu and Dupuy (2022)
Treatment outcome	SVM	FOLFOXai signature identifies mCRC patients for whom oxalilplatin-containing therapies are less beneficial	Abraham et al. (2021)
Drug repurposing	SVM	Molecular simulation with approved drugs to identify molecules with RET inhibition profile	Ramesh et al. (2022)
Prognostic factors	LVM	Identification of somatic oncogenic mutations	Liu Y et al. (2022)
Prediction of benefits	Decision tree	Mutation signature predictive of the benefit of immunotherapy in NSCLC	Liu Z et al. (2022)
Efficacy predictors	SVM Regression	Prediction of the efficacy of anticancer drugs based on clinical and molecular features of OSCC	Brindha et al. (2022)
Toxicity predictors	RF	Identification of SNPs in the PI3K/AKT pathway associated with toxic effects during chemotherapy in LACC patients	Guo et al. (2023)
Multi-omics data integration	RF	Prediction of tumor recurrence and survival in PDAC patients based on multi-omics data from metastatic and non-metastatic microbiome patient signatures	Li S et al. (2022)
Medical imaging (radiomics)	Regression	Prediction of OS and PFS in patients with ESCC based on CT image radiomics signatures	Cui et al. (2022a)

Abbreviations: CRC, colorectal cancer; CT, computer tomography; ESCC, esophageal squamous cell carcinoma; FOLFOXai, folinic acid, fluorouracil, oxaliplatin artificial intelligence; LACC, locally advanced cervical cancer; LVM, latent variable model; mCRC, metastatic colorectal cancer; NN, neural network; NSCLC, non-small cell lung carcinoma; OS, overall survival; OSCC, oral squamous cell carcinoma; PDAC, pancreatic ductal adenocarcinoma; PFS, progression free survival; RF, random forest; SAR, structure-activity relationship; SNPs, single nucleotide polymorphisms; SVM, support vector machine.

In unsupervised learning, ML methods are not task-specific (i.e., they are not based on a specific predicted outcome, such as survival), provide general insights, and include methods such as k-means clustering and hierarchical clustering. These methods have been used in oncology to identify cancer subtypes, stratify patients, and create clusters from gene expression data to identify patterns and groupings (Eckardt et al., 2023). Another example of unsupervised learning is the latent variable model (LVM), which can capture unobserved variables that may affect the outcome. LVM can therefore be used to regress variables into one or more classes that would best explain the heterogeneity in the data (Miettunen et al., 2016).

Supervised methods include the support vector machine (SVM), which divides data into categories (two or more) to solve both regression and classification problems. It is based on kernel algorithms that can expand the feature space to make data more accessible. It is considered one of the most robust models to date (Tate et al., 2006; Pacurari et al., 2023).

Decision tree learning is a supervised model that is commonly used in clinical practice for decision-making processes. In this case, ML can help identify which variables have the highest separability to the desired categories and is used for both classification and regression (Podgorelec et al., 2002). Random Forest (RF) is an extension of decision tree learning that combines multiple randomly generated decision trees to improve decision making. Given the complexity of biology, data scientists usually prefer RF to decision tree (Breiman, 2001; Cui et al., 2022b).

Finally, neural networks (NNs) are models used in all three types of learning. NNs, which mimic the neural architecture of the human brain, can integrate multiple sources of information and process them in nodes and layers. Each node represents a specific feature with a specific weight, and nodes of the same level represent a layer. Moreover, nodes of different layers can be connected to each other. If the information stored in the node is valuable, the node weight exceeds a certain threshold, which means that the node is triggered and the network is active. During the training, the weight values and threshold are continuously adjusted to form the best combination of nodes and weights that results in the most informative NN (Kriegeskorte and Golan, 2019).

Deep Learning (DL) is one of the NN algorithms where the number of hidden layers and nodes is increased and the overall size of the network is very large, which allows better representation of complex relationships. The main advantage of DL is that it identifies hidden features as part of the learning process, making DL faster and more automated compared to ML (Erickson et al., 2017). These features also correlate with sensitivity and availability of cheaper computing power. Therefore, DL is now referred to as a specific subset of ML with its own algorithms and applications, and has become one of the most widely used approaches in cancer research. Thus, in the following part of this section, we discuss some applications of DL in cancer research.

The use of DL in oncology began with the analysis of medical images, because it is particularly good at identifying pathogenic features of the observed cells, and in certain cases the performance of DL is almost equal to human performance (LeCun et al., 2015; Jalloul et al., 2023). For example, the application MIA was developed to analyze images from microscopy and can be used for classification, object recognition, segmentation, and tracking (Körber, 2023). In addition, medical images of histopathological tumor sections were used to test whether DL can predict response to therapy in patients with adenocarcinoma of the gastroesophageal junction. In this work, researchers found that DL is able to distinguish patients who respond to neoadjuvant chemotherapy from those who do not by extracting certain features on the images before therapy initiation (Hörst et al., 2023). A Swedish study has developed a DL tool for detecting lymph node metastases in colorectal cancer that has excellent accuracy compared to human performance. This tool reduces the time required to assess lymph nodes, which in turn improves the diagnostic process and treatment decisions (Kindler et al., 2023). Another DL tool has been developed to assist clinicians in digital pathology by assessing the tumor cellularity of histopathologic hematoxylin and eosin sections (Altini et al., 2023). Apart from the importance that this algorithm may have in the clinical setting, it is important to point out that its use may also be useful in research, as it allows pathologists to share valuable information with researchers in an automated manner. A high percentage of tumor cells is an important requirement for researchers to perform NGS sequencing, as the biological material taken from the slice must be representative of the tumor in order to reduce the contribution of normal adjacent tissue, thereby reducing background noise and improving sequencing quality. In addition, DL has been successfully developed to predict optimal radiotherapy for patients with brain metastases using CT images and non-image clinical information (Cao et al., 2023). It has also been developed to predict pneumonitis risk in lung cancer patients treated with immune checkpoint inhibitors and to identify morphologic features that predict *ERBB2* status and trastuzumab efficacy in breast cancer patients (Bychkov et al., 2021; Cheng et al., 2023). In a retrospective multicenter study, a DL algorithm was developed to help radiologists diagnose breast cancer lesions and differentiate axillary lymph node metastases based on radiological features (Zhou et al., 2023).

Although medical imaging remains the foremost application of DL, it is also used in the analysis of genomics and transcriptomics data, including data from single-cell experiments that can improve variant detection calling at cell-specific resolution. DL improvements in single-cell sequencing could enhance the ability of researchers to understand intratumoral heterogeneity and identify previously unknown cell subpopulations, making this a particularly attractive area for molecular oncology (Erfanian et al., 2023; Halawani et al., 2023; Shen et al., 2023). DeepTTA is a DL model that uses transcriptomic data to predict anticancer drug response, which can shorten the preclinical phases of drug development and drug screening. In addition, DL models which can predict cancer drug response can also be used to identify new potential clinical applications of known drugs based on target affinity and mechanism of action (Douglass et al., 2022; Jiang et al., 2022; Park et al., 2023). DeepTAP is another DL algorithm capable of predicting sequence peptides that bind to tumor neoantigens, which may be of interest to the field of cancer immunotherapy (Zhang X et al., 2023). With this in mind, DL algorithms are being used in precision medicine to predict anticancer drug response in patient-derived cancer cell lines, as in the case of the DeepDRK framework, which is freely available (Wang Y et al., 2021). A method based on DL was developed to study the uptake of targeted nanoparticles in triple-negative breast cancer, which could be useful for proper dosing in clinical practice (Ali et al., 2022). A nanodiamond biosensor platform using DL was developed to rapidly assess individual specific sensitivity to oxidative phosphorylation inhibitors in patients with hepatocellular carcinoma (Xu et al., 2023).

Finally, network pharmacology is a new approach in drug development that aims to understand the network interactions of multiple drug combinations. In this context, the network algorithms of DL may be useful to identify synergistic combinations of multiple drugs targeting a specific network that can be used to improve cancer treatment (Noor et al., 2023). DeepDTnet, for example, is a DL method for network-based target identification that reveals novel therapeutic effects of known molecules, which in turn can accelerate drug repurposing, a process aimed at finding new uses for drugs that are approved or in trials (Zeng et al., 2020). The strategy of drug repurposing offers numerous advantages over developing a completely new drug, such as a lower risk of failure because safety and risk assessment have already been tested, cost and time savings in the preclinical phases, and finally, phase I and II results are already available, thanks to sophisticated algorithms such as those used in DL and ML (Pushpakom et al., 2019). Based on this approach, many oncology and non-oncology drugs have been reviewed in recent years, and drug repurposing is particularly valuable in rare or late-stage diseases where the development of a new drug may be difficult in terms of patient recruitment and the time required for a complete clinical trial may be unreasonable.

In PGx studies, DL has also been used to predict the toxicity of specific medications. In recent works, DL methods were able to predict the toxicity of radiation-based therapy in four different cancer types (Tan et al., 2023) and identify SNP signatures associated with urinary symptoms and overall toxicity in prostate cancer patients treated with radiation therapy (Massi et al., 2020). An important implementation of DL in PGx studies may interest medical imaging with feature extraction that predicts drug response based on SNP signatures. However, this task is very difficult to accomplish because the DL algorithms used to scan medical images are designed to extract “abnormal” features, and PGx often refers to germline (i.e., “normal”) variants. However, in this way, it would be possible to reduce the number of diagnostic tests a patient has to undergo, also in the context of the most appropriate choice of therapy after diagnosis.

The use of DL, as well as ML in general, can improve healthcare and assist clinicians by shortening the time required for diagnosis and staging and facilitating decision-making in drug selection and administration of the correct dose, thus contributing to the clinical translation of precision medicine in cancer.

### 4.3 Machine learning in PGx studies

PGx studies have gradually shifted from reactive testing on a single gene to proactive testing on multiple genes to improve treatment outcomes. This move has been made possible by the implementation of high-throughput data generation and analysis.

With the advent of ML and computer science in cancer research, it is now possible to discover previously unknown cancer-related features and latent signatures that impact tumor development, progression and recurrence. In addition, ML offers the opportunity to gain insight into failed clinical trials to understand their limitations and potential benefits, and to prevent toxicity and other drug effects that can impact patient quality of life and treatment efficacy (Harrer et al., 2019).

One of the first constraints in screening new drugs is selecting candidate molecules from the initial bulk of drug libraries. By combining genomic features of cell lines and chemical information of molecular compounds, researchers have been able to create *in silico* ML multi-drug models to predict IC_50_ values, saving cost and time (Menden et al., 2013). Furthermore, these *in silico* approaches enabled the identification of genomic events associated with altered drug sensitivity, optimizing drug trial design (Huang et al., 2017).

In cancer, multitherapy is often used not only to reduce the toxicity of a single anticancer agent and achieve synergistic effects, but also to overcome drug resistance (Dear et al., 2013; Lee et al., 2019; Kim et al., 2020). Screening to predict synergistic drug combinations is a computational approach that has been explored using ML technology. For example, screening multiple administrations of over 40 different drugs in melanoma cancer cells led to the identification of 11 validated, previously untested drug combinations that lead to different outcomes (Gayvert et al., 2017).

As mentioned earlier, there is growing evidence that optimal prediction of drug response relies on individualized molecular profiling (Bode and Dong, 2017). Many ML approaches in PGx studies have been developed to predict the best match between genetic alterations involved in the pathogenesis or recurrence of a given cancer and drugs targeting these alterations (Chang et al., 2018). From this perspective, therapeutic drug monitoring (TDM) is an experimental procedure that measures the plasmatic concentration of a given drug in a specific time window after administration. Recent work has shown that ML methods applied to TDM were able to predict the appropriate dosing for various drugs, e.g., lapatinib dose for patients with metastatic breast cancer (Yu et al., 2022) and cisplatin dose in cohort of patients with head and neck cancer to avoid cisplatin-related toxicity (Cauvin et al., 2022).

### 4.4 Multi-omics integration, ML and PGx

Integration of different omics information better captures the complexity of cancer through different molecular layers and therefore improves diagnosis, prognosis and treatment compared to using a single “omic” alone (Heo et al., 2021).

There are many examples of successful integration of multi-omics approaches. Whole genome and transcriptome data have been used to quantify the extent of specific genetic alterations at the mRNA level and derive quantitative trait loci (eQTLs). In this context, polymorphisms associated with specific phenotypes are found to be associated with eQTLs, and genetic risk factors associated with eQTLs can *bona fide* predict the level of the corresponding gene product (Nicolae et al., 2010).

Epigenomics and transcriptomics/proteomics data can be aligned to explain how epigenetic changes can affect protein turnover (Wang X et al., 2021). In addition, transcriptome sequencing has often been combined with miRNome sequencing to determine which microRNAs and non-coding RNAs may alter gene expression and modulate response to chemotherapy (Fazi et al., 2015; Cuttano et al., 2022). Many consortia and catalogs have been developed to promote the understanding of tumor processes with high-throughput data, such as the Clinical Proteomic Tumor Analysis Consortium (CPTAC), which integrates genomic and proteomic data to create a proteogenomic portrait of cancer toxicity and resistance (Ellis et al., 2013), the International Cancer Genome Consortium (ICGC), which provides cancer genomic, DNA methylation and gene expression data (Zhang et al., 2011), and The Cancer Genome Atlas (TCGA) program, which provides a collection of genomic, epigenomic, transcriptomic and proteomic data on 33 different cancer types (The Cancer Genome Atlas Research Network et al., 2013). These data collections are the tip of the iceberg of the various omics data consortia that have emerged to date, and they serve as a valuable resource for omics and ML modeling studies of cancer.

The application of ML with the integration of multi-omics data has resulted in several scores for risk prediction and diagnostic/therapeutic potential, such as the polygenic risk score. The polygenic risk score considers all genetic inheritance variants known to be associated with a particular disease and measures the risk associated with the development of the disease under investigation, thereby improving risk stratification and screening (Akdeniz et al., 2023). Other examples include the BRECADA application, which uses genetic and nongenetic risk factors for early detection of breast cancer, and the OncoNPC signature, which classifies cancer of unknown primary and accordingly tailors initial palliative treatment intent, a strategy that often leads to better patient outcomes compared with cancer treated without querying the OncoNPC signature (Moon et al., 2023; Tao et al., 2023). In addition, some radiomics features have been extracted from images of brain metastases of extracranial primary tumors and correlated with the expression level of PD-L1, allowing stratification of patients according to their sensitivity to immune checkpoint inhibitors using a ML noninvasive classifier (Meißner et al., 2022).

The ability of ML to handle multiple data structures, namely, clinical, molecular and imaging data, allows to discover hidden correlations among different input data in PGx studies to make more accurate predictions and inferences. However, there are several crucial aspects to consider when managing and using multi-omics data that should be examined. First, collection of multi-omics data requires careful evaluation of the entire experimental workflow, from tissue collection and high-quality extraction of nucleic acids and proteins to sample preparation and sequencing. Second, data analysis pipelines need to be developed to integrate individual omics approaches. In this context, early, intermediate and late ML fusions help to address the management of multimodal approaches with positive impact on clinical cancer research. Third, the expertise required for analytical and bioinformatics analyses often requires the collaboration of multiple experts to properly mediate the integration of multi-omics data. Thus, building a multidisciplinary team is therefore challenging for the success of multimodal data integration, but the positive outcomes of multimodal approaches have already been demonstrated and adopted (Kwon et al., 2015; Jing et al., 2020).

### 4.5 Use of ML in clinical trials

Clinical trials are later phases of drug development and incur very high costs. Clinical trials in oncology have the highest overall failure rate, mainly due to poor trial design (Wong et al., 2019). Therefore, the use of ML in clinical trials could be an opportunity to increase success. However, most applications of ML have focused on preclinical studies rather than improving clinical trial design, possibly due to the significant regulatory challenges associated with the use of ML in a clinical context (Massella et al., 2022).

ML improvements in study design can be attributed to three main strategies: cohort composition to improve suitability by reducing cohort heterogeneity, patient recruitment to improve eligibility by maximizing patient-study match, and patient monitoring to improve adherence and endpoint detection to reduce dropout rates (Harrer et al., 2019; Van Der Lee and Swen, 2023).

As mentioned earlier, oncology clinical trials often fail to meet primary endpoints due to inadequate stratification criteria, poor recruitment and evidence of severe drug toxicity (Hwang et al., 2016; Kim et al., 2023). To address these issues, the RainForest algorithm was developed. The CAIRO2 clinical trial investigated the use of cetuximab in patients with metastatic colorectal cancer and concluded that there was no benefit to using this agent in the overall population. However, the RainForest algorithm was able to identify a small subset of patients who actually benefit from cetuximab treatment based on the SNP germline profile of patients (Ubels et al., 2020). The use of the RainForest algorithm in clinical trials can save enormous resources, as the cost of a single agent is estimated to be around 2.8 million US dollars in the final stages of approval (DiMasi et al., 2016).

Severe toxicity is another major issue in clinical trials, and changes or interruptions to treatment schedules account for at least 30% of failures in phases II and III (Harrison, 2016). ML has also supported the design of clinical trials in term of drug safety. A recent work has shown that SNPs signatures can serve as genetic predictors of toxicity in personalized medicine. The germline variant rs4864950 T>A in the *KDR* gene increased the risk of composite toxicity (occurrence of any of hypertension, diarrhea and dermatological reactions) in patients treated with the VEGFR TKIs sorafenib and regorafenib (Quintanilha et al., 2022a). In another work, the *ABCB1* rs9282564 was the variant most strongly associated with hypertension and nonhematological toxicities in ovarian cancer patients treated with bevacizumab, and SNPs in genes related to the biological oxidation pathway (*CYP3A4* rs28371763 and *CYP1B1* rs9341266) were the most significant variants associated with hematological toxicity in the same cohort (Polano et al., 2023).

Clinically relevant predictors of toxicity have also been found in many GWAS studies, e.g., SNPs predicting severe skin toxicity in patients with colorectal carcinoma treated with cetuximab (Baas et al., 2018) or predicting dysphagia in patients with nasopharyngeal carcinoma treated with radiotherapy (Wang et al., 2022) or predicting neurotoxicity and leukoencephalopathy in patients with lymphoblastic leukemia treated with methotrexate (Bhojwani et al., 2014). Many other correlations between SNPs and toxicity can be found in the literature. Most importantly, prediction of cancer-related toxicities can prevent deterioration in patients’ quality of life and adherence to treatment and can be used to manage chemotherapy-related adverse effects in the clinical setting (On et al., 2022).

### 4.6 Challenges of ML in PGx

Incorporating assessment of somatic and germline variations into treatment decisions with FOLFIRI in elderly patients with metastatic colorectal cancer has been shown to be effective. This regimen requires assessment of *RAS* mutations as well as *DPYD* and *UGT* polymorphisms prior to treatment with the FOLFIRI protocol (Mathijssen et al., 2003; Morel et al., 2006; Sepulveda et al., 2017; Battaglin et al., 2018). Although PGx test guidelines have already been implemented in clinical practice, another important issue in this context is the implementation of ML in clinical practice. ML has demonstrated its usefulness in retrospectively classifying patients during clinical trials to assess drug safety and prognosis (Chang et al., 2018; Quintanilha et al., 2022a; Chen et al., 2022), but the incorporation of ML into clinical trials and clinical practice is still up for debate. Standard guidelines and protocols need to be thoroughly regulated to obtain comparable information and a precise methodological approach, not only for the algorithms of ML, but also for PGx studies. It is worth noting that different ML models can be applied to the same given subject, as there is no universal applicability of ML algorithms and this could lead to different results in the same dataset.

Moreover, NGS has only recently been introduced into clinical practice to assess diagnosis and prognosis and to evaluate therapeutic strategies, but it is still a niche and there is room for improvement. In addition, sequencing of some parts of the genome remains challenging, e.g., highly polygenic regions, pseudogenes, triplet expansions, low complexity regions, short repetitive sequences, regions of high-similarity, and complex structural rearrangements (Treangen and Salzberg, 2012; Rojahn et al., 2022). It is estimated that approximately 14% of clinically relevant genetic tests are located in these genomic regions (Lincoln et al., 2021), and correct variant identification can be difficult. On the one hand, short tandem repeats and complex structural variants known to play a role in the pathogenesis of certain diseases can now be sequenced using a targeted approach with long reads sequencing technology, as longer reads are expected to generate appropriate sequence length that overlaps better during assembly (Stevanovski et al., 2022). On the other hand, these technical difficulties can be at least partially overcome by adapting NGS analysis workflows accordingly (Rojahn et al., 2022). However, detecting variants in these regions remains challenging and difficult to validate. Detection of rare and very rare mutations with low allele frequency should also be considered. Inclusion of all probes in the ML learning datasets can be helpful, because some isoforms are more informative than others, and pooling them into averages may lead to dilution or loss of this information. NGS and ML can lead to great improvements in PGx studies, as multiple samples and multiple genes can be tested simultaneously, and clinically relevant hidden patterns can be uncovered from complex data structures.

However, ML and DL also have their own limitations. In general, ML algorithms are not error-free: one of the biggest challenges is the learning model itself. Indeed, many ML approaches have problems with underfitting or overfitting, where the data follows the trained data or noise signals too closely, resulting in poor curve estimation. An appropriate size of the training dataset is also important for ML models to learn properly and make accurate predictions. The reliance on large datasets for developing accurate models is also a challenge due to the lack of sample availability. In addition, the relationship between bias and variance also plays a role in obtaining the best performance from ML models. On the other hand, the accessibility of the code used for ML in PGx studies is low (Huang et al., 2017). To further improve knowledge and sharing among distant researchers around the world, platforms for sharing data and code should be established. To this end, computer scientists could have the opportunity to address underestimated problems and find common resources to overcome them. In addition, the development and improvement of multimodal ML methods, such as late ML fusions, may encourage a more holistic view of specific patient characteristics across different input data types.

As previously reported, PGx testing has demonstrated its utility in many situations, and new variants of uncertain significance have been reported thanks to GWAS and NGS PGx studies. On the one hand, the impact of these new variants on PGx testing is still being evaluated. On the other hand, the clinical implementation of genotyping of genes known to be involved in individual drug response needs to be monitored. In particular, the development of genotyping panels should also be improved to enable the translation of new relevant findings into the clinical settings. One of the most important and unanswered questions related to the use of these genotyping panels is how representative they are of individual variability in drug response. Moreover, PGx studies often suffer from a lack of homogeneous tumor samples, which is particularly true for rare tumors and inconsistent sample ancestry origins and incomplete data are also common complaints. Harmonization of samples and data collection, as well as free and easy access to sample datasets, could facilitate PGx studies with ML. Finally, new and standardized scores to track NGS quality, ML accuracy and significance of PGx variants could also be developed to address new tasks.

## 5 Conclusions and future prospective

The molecular revolution in oncology continues to grow, with the paradigm flowing from pathological oncology based on morpho-histological assessment of tumor specimens to molecularly driven oncology, where precise individual molecular features are considered as part of diagnosis, grading and prognosis to tailor treatment to the individual. In this context, not only are targeted molecular therapy and patient genetic characteristics important factors in predicting therapeutic response, but drug repurposing can also be a valuable resource by using drugs approved for other diseases to treat cancer.

The therapeutic margin in cancer treatment is often small because the dose-toxicity curve is often close to the dose-response curve, so even small fluctuations in drug concentration can lead to severe side effects (Lowe and Lertora, 2012). Therapeutic drug monitoring (TDM) has already demonstrated its validity for assessing correct dosing, although its application is still limited to a small number of anticancer drugs (Knezevic and Clarke, 2020). Assessing genetic polymorphisms that may alter drug response can be beneficial for many reasons, including drug safety profile, patient adherence, and cost savings, not only in oncology, where PGx testing has been adopted more rapidly, but also in other areas (Roden et al., 2019). Therefore, proactive testing is becoming increasingly important for developing treatment strategies for patients based on individual genetic variability and needs, from the perspective of even more personalized medicine.

ML can improve understanding of data generated from PGx studies, increase understanding of clinical trial results, predict clinical outcomes, and discover new biomarkers even at very early stages of drug development to identify subgroups of patients who actually benefit from treatment, and subgroups of patients who do not benefit and may experience toxicity. Although the results of ML models derived from high-throughput data should be confirmed by classical functional studies, they offer researchers the opportunity to explore the extensive relationships that exist in biological processes.

Finally, ML can also be used in cancer theranostics, a combination of diagnostic and therapeutic procedures in which radioactive drugs are first used to identify the disease and then to deliver therapies. ML is a very innovative and versatile tool but the adoption of ML into routine clinical practice is still unsettled and does not yet seem to be truly welcome. On the one hand, this feeling can be explained by the lack of standardization and the fact that specific guidelines for the use of ML have not yet been established. On the other hand, the lack of understanding of the hidden algorithms driving ML decisions may be perceived as a barrier to clinicians’ skills and expertise. In addition, there is no single ML model that can solve a particular problem, so the use of a particular model is not tailored to the task at hand, which may increase the risk of complications in ML harmonization. Finally, patients should be informed and their data protected. Therefore, ethical aspects related to the security of individual data and the protection of privacy are a challenge and a mandatory requirement to gain patients’ trust and prevent them from feeling threatened by ML. The innovation that the use of ML could bring to clinical practice is unquestionable and could help to improve cancer treatment towards a more personalized medicine.
